# Histopathological Characterization of Tail Injury and Traumatic Neuroma Development after Tail Docking in Piglets

**DOI:** 10.1016/j.jcpa.2016.05.003

**Published:** 2016-07

**Authors:** D.A. Sandercock, S.H. Smith, P. Di Giminiani, S.A. Edwards

**Affiliations:** ∗ Animal and Veterinary Science Research Group, Scotland's Rural College (SRUC), West Mains Road, Edinburgh, UK; † Royal (Dick) School of Veterinary Studies and the Roslin Institute, Easter Bush Campus, Roslin, Midlothian, UK; ‡ School of Agriculture, Food and Rural Development, Newcastle University, Newcastle upon Tyne, UK

**Keywords:** pain, pig, tail docking, traumatic neuroma

## Abstract

Tail docking of neonatal pigs is widely used as a measure to reduce the incidence of tail biting, a complex management problem in the pig industry. Concerns exist over the long-term consequences of tail docking for possible tail stump pain sensitivity due to the development of traumatic neuromas in injured peripheral nerves. Tail stumps were obtained *post mortem* from four female pigs at each of 1, 4, 8 and 16 weeks following tail amputation (approximately two-thirds removed) by a gas-heated docking iron on post natal day 3. Tissues were processed routinely for histopathological examination. Non-neural inflammatory and reparative epidermal and dermal changes associated with tissue thickening and healing were observed 1 to 4 months after docking. Mild neutrophilic inflammation was present in some cases, although this and other degenerative and non-neural reparative changes are not likely to have caused pain. Traumatic neuroma and neuromatous tissue development was not observed 1 week after tail docking, but was evident 1 month after tail docking. Over time there was marked nerve sheath and axonal proliferation leading to the formation of neuromata, which were either localized and circumscribed or comprised of multiple axons dispersed within granulation tissue. Four months after tail resection, neuroma formation was still incomplete, with possible implications for sensitivity of the tail stump.

## Introduction

Tail docking (amputation of the distal part of the tail) is often carried out in commercial pig production on neonatal piglets within the first few days of life in order to reduce the subsequent risk of tail biting, which represents a major health and welfare issue that can cause significant production losses (Hunter *et al.*, 2001). The formation of traumatic or ‘amputation’ neuromas as a consequence of tail docking has been reported previously in dogs (Gross and Carr, 1990), lambs (French and Morgan, 1992) and pigs (Simonsen et al., 1991, Herskin et al., 2015) and occurs as a result of repair processes in injured peripheral nerves in the tail (Foltán *et al.*, 2008). Traumatic neuromas are defined as non-neoplastic proliferations of epineurial, perineurial and endoneurial connective tissue, Schwann cells and regenerating cells representing attempts at regeneration (Swanson, 1961).

It has been suggested that neuroma formation following tail docking may cause detrimental sensory changes in the tail due to altered peripheral nerve activity that may cause pain or chronic discomfort (Simonsen *et al.*, 1991). Following peripheral nerve injury, it has long been recognized that regenerating endings of afferent sensory fibres can become trapped within scar tissue and produce spontaneous or abnormal neuronal firing (Govrin-Lippmann and Devor, 1978, Devor, 1983). This may lead to changes in peripheral sensitivity to mechanical and thermal stimulation, manifesting as altered sensations that range from anaesthesia, paraesthesia, dysaesthesia (unpleasant abnormal sensation) to pain (Holland and Robinson, 1998, Rajput et al., 2012). However, the reported incidence of these clinical presentations in human patients following surgical amputation of digits and toes is relatively low, ranging from 2.7% (Fisher and Boswick, 1983) to 7.8% (Van der Avoort *et al.*, 2013). In general, where post-amputation healing occurs without complications, the resulting neuroma is not normally painful *per se*. When clinical signs indicative of pain or discomfort are apparent they tend to be attributable to coexistent scar tissue, abscess, haematoma or osteomyelitis (Beggs, 1997).

There have been a few histopathological studies of traumatic neuromas in pig tails (Simonsen et al., 1991, Done et al., 2003, Herskin et al., 2015). These have focused mostly on superficial descriptive histological analysis of the neuroanatomical features of the peripheral nerves in the pig tail at the time of slaughter, with respect to different methods of docking. To date there has been no attempt to characterize in depth the general histopathological changes in the tail (including changes in the peripheral nerves) over the animal's life time after tail docking.

The aim of this preliminary study was to examine the effect of commercial tail docking by hot iron cautery in neonatal pigs on histopathological changes in tail anatomy, with specific attention to the onset and progression of traumatic injury, healing and neuroma formation over time.

## Materials and Methods

### Animals and Housing

Sixteen female piglets, *Sus scrofa domesticus* (Landrace/large white × synthetic sire line) were used from a commercial herd reared at Cockle Park Farm, Newcastle University. The piglets were obtained from four separate litters that were reared in loose-housing farrowing pens until they were weaned at 28 days and then transferred to conventional fully slatted weaner, grower and then finisher pens until they were removed for humane destruction and subsequent post-mortem tissue collection. The pigs were selected randomly from healthy commercial stock, having been monitored on a daily basis by farm staff. At the time of death, all pigs appeared clinically sound.

### Experimental Procedures

All procedures on animals were in accordance with institutional guidelines and UK animal welfare regulations, and the study was conducted where practicable in accordance with ARRIVE guidelines (Kilkenny *et al.*, 2010).

**Fig. 1:**
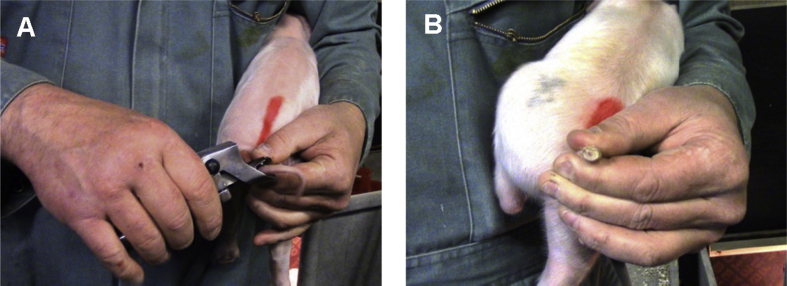
Piglet tail (A) immediately before and (B) after approximately two-thirds removed by hot iron docking.

Piglets were tail docked (approximately two-thirds of the tail removed) on post natal day 3 using a gas-heated docking iron (East Riding Farm Services, Driffield, UK) in line with commercial pig management procedures (Fig. 1). All tail tips healed without complications and did not incur any further injury throughout the duration of the study.

### Sedation and Humane Killing

At the time of humane killing, groups of four pigs were moved in an animal transport trailer a short distance from the home pen building to holding pens located within a nearby surgical and post-mortem facility. The pigs were weighed individually to determine the required drug dosages. For sedation, the pigs received an intramuscular injection in the neck of ketamine (5 mg/kg, Vetoquinol, Buckingham, UK), midazolam (0.5 mg/kg, Hameln, Gloucester, UK) and medetomidine (10 μg/kg, Vetoquinol) and were left undisturbed under dimmed light conditions for 10–15 min. Once each pig was sedated (i.e. immobile, absence of reaction to touch and human presence) the ear vein was catheterized. Pigs were killed humanely by injection of sodium pentobarbitone (150 mg/kg intravenously; Abbott Laboratories, Abbott Park, Illinois, USA). Death was confirmed by respiratory arrest and loss of corneal reflex. All pigs were exsanguinated by cutting the jugular and carotid arteries prior to post-mortem tissue collection.

### Collection and Preparation of Tail Stumps

Following humane killing and exsanguination, the dorsal surface of the tail was marked with an indelible marker pen to aid tissue orientation during processing. Approximately 2 cm of the distal tail tip was cut off with a scalpel. After the tail portion was removed it was further transected through the midsagittal plane to aid tissue fixation. One-half of the bisected tail sample was fixed in 10% neutral buffered formalin for a minimum of 5 days and then transferred into 14% EDTA (pH 7.4) for 7–9 days before routine processing and embedding in paraffin wax. Two left side and two right side tail samples were used for each post-docking group (*n* = 4).

The tail samples were embedded so that the tissue could be sectioned longitudinally lateromedially. Serial sections (>200) were cut at 4 μm and every tenth section, up to a maximum of 15 sections per block was stained with haematoxylin and eosin (HE). For each of these, the next consecutive section was also retained. The HE-stained slides were examined microscopically to determine if sections contained peripheral nerve tissue. The consecutive section containing the most neural tissue was then labelled immunohistochemically to determine expression of S100 for visualization of peripheral nerves (Dahl-Pedersen *et al.*, 2013).

### Immunohistochemistry

Briefly, serial sections were cut from each block, dried overnight at 37°C and incubated at 60°C for 25 min prior to dewaxing in xylene, hydrating in ethanol, washing in water and then washing in Tris buffer. No antigen retrieval is required for this method. Each section was incubated for 30 min at room temperature (RT) with a 1 in 400 dilution of rabbit anti-S100 polyclonal antibody (Dako Z0311, Dako, Ely, UK). Dako antibody diluent was used (Dako S0809). Endogenous peroxidase was blocked using Dako REAL™ peroxidase blocker (Dako S2023). Secondary detection was achieved using goat anti-rabbit horseradish peroxidase conjugate (Dako P0448) diluted 1 in 50 for 30 min at RT. Visualization was achieved using 3, 3′ diaminobenzidine (DAB, Dako K3468) liquid DAB^+^ substrate chromogen system. Nerve processes in pig small intestine served as positive controls and pre-existing axons in the pig skin acted as internal controls. Negative controls consisted of antibody diluting fluid only, with no primary antibody added.

### Histopathological Scoring

Examination of all sections was completed by one person (SHS) who was blinded to the time point after tail docking. A semiquantitative scoring scheme was used, where the feature was recorded as either absent or present, then classified on a three-point categorical scale of relative abundance: +, low; ++, medium; and +++, high (see Supplementary data). The histopathological features scored were based on a list of observations previously reported by Done *et al.* (2003) and expanded on as part of this study.

### Statistical Analysis

All statistical procedures were performed using Sigma-Plot 11 (Jandel Inc., Richmond, California, USA). Comparisons of the prevalence of an observed pathological feature at different times after tail docking were conducted using Fisher's Exact test. Categorical abundance scores for each histopathological feature were compared for different times after tail docking using the Mann–Whitney *U* test. Results were considered significant at *P* <0.05.

## Results

Data values representing the number of pigs at each time point after tail docking exhibiting a certain pathological feature and associated maximum abundance score and statistical analyses are shown in Table 1.

### Histopathology 1 Week after Tail Docking

**Fig. 2:**
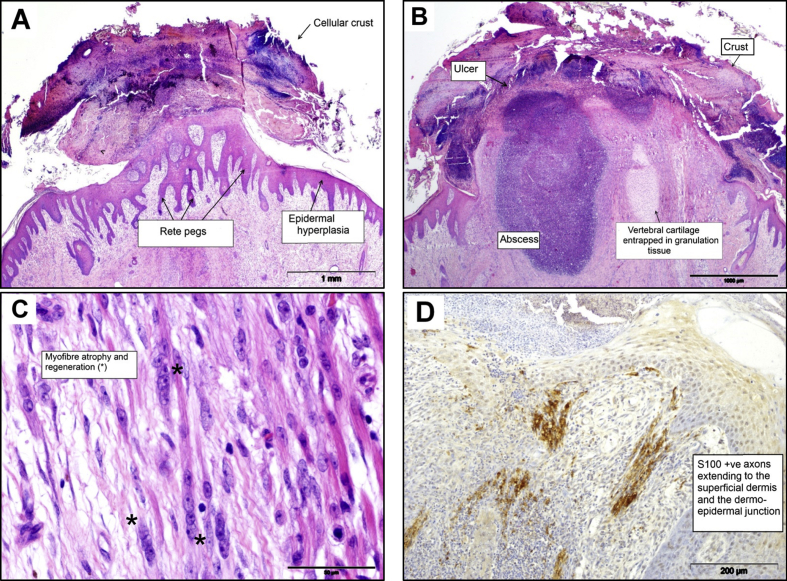
Histopathological features in sections of pig tail stump 1 week after docking. (A) Cellular crust, epidermal hyperplasia and accentuation of rete pegs. HE. (B) Widespread granulation tissue, neutrophilic inflammation with some ulceration, abscess formation and oedema in the dermis. HE. (C). Myofibre atrophy and regeneration (*) in the deep skeletal muscle around the coccygeal vertebrae. HE. (D) De-novo axonal growth extending to the superficial dermis and dermo-epidermal junction (S100 expression). IHC.

Tail tips at the site of injury were fully covered with a surface crust (eschar) on gross examination. The surface crust, comprising of necrotic cell debris and serous fluid, was evident at the edge of the incision in all four tails examined by histology and many bacterial colonies were buried within it. In the epidermal layers, varying degrees of epidermal hyperplasia were observed in all tails, along with anastomosing rete pegs and orthokeratotic hyperkeratosis (Fig. 2A). Parakeratosis was noted in one tail and, in two of four tails, there were foci of epidermal erosion. In some tail sections, spongiosis, subcorneal and intra-epidermal pustules were observed, although the latter were rare. Full re-epithelialization had only occurred in one of the four tails. In the dermis there was widespread granulation tissue, areas of fibroplasia, and neutrophilic inflammation with abscess formation in one tail (Fig. 2B). The presence of mild dermal oedema was significantly greater (*P* <0.05) at this stage post docking. Sporadic angiogenesis unassociated with granulation tissue was also seen. The presence of mild to moderate myofibre atrophy and regeneration in the deep skeletal muscle around the coccygeal vertebrae was also observed at this stage post docking (Fig. 2C). There was evidence of mild bone remodelling in two of the four tails. Using the S100 neurofilament stain for the identification of peripheral nerves, there was no evidence of traumatic neuroma formation at this time point, although there was ‘de-novo’ axonal growth extending to the superficial dermis and dermo-epidermal junction (Fig. 2D).

### Histopathology 4 Weeks after Tail Docking

**Fig. 3:**
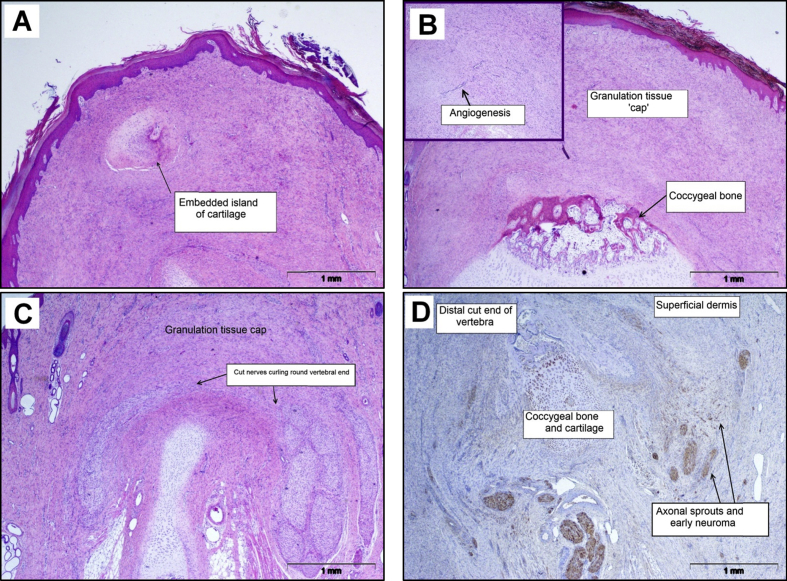
Histopathological features in sections of pig tail stump 4 weeks after docking. (A) Full re-epithelization of tail tip, mild epidermal hyperplasia, orthokeratotic hyperkeratosis and parakeratosis (note the island of cartilage in granulation tissue). HE. (B) Mature granulation tissue cap in the distal tip at the site of injury in the dermis. HE. (C) Widespread axonal proliferation and infiltration of the superficial dermis limited by a granulation tissue ‘cap’. HE. (D) Neuromatous tissue/early neuroma formation with newly formed axonal endings following a course of attempted re-innervation around the cut vertebral end, proximal to the granulation tissue (S100 expression). IHC.

Tail tips were fully healed on gross examination in terms of epidermal integrity, as full re-epithelization was observed in all four tails (Fig. 3A). There was mild to moderate epidermal hyperplasia, orthokeratotic hyperkeratosis and parakeratosis evident in three of four tails. Mild intra-epidermal pustule formation was observed in one tail, but subcorneal pustules were not observed. In the dermis the formation of a prominent mature granulation tissue ‘cap’ was observed in the distal tip at the site of injury (Fig. 3B). This was characterized by extensive dermal fibroplasia and angiogenesis that extended to the transected coccygeal vertebra. Remnants of coccygeal cartilage were observed embedded in the granulation tissue of one tail (Fig. 3A). Mild dermal neutrophilic inflammation was evident in two of four tails, but no dermal oedema was present. Some coccygeal myofibre atrophy and regeneration were observed, but there were no signs of osteomyelitis or bone remodelling. S100 neurofilament immunolabelling highlighted widespread axonal proliferation and infiltration of the superficial dermis, although this was limited by the granulation tissue cap at this time point after docking injury (Fig. 3C). Neuromatous tissue/early neuroma formation was observed in two of four tails, characterized by newly formed axonal endings following a course of attempted re-innervation around the cut vertebral end, proximal to the granulation tissue (Fig. 3D).

### Histopathology 8 Weeks after Tail Docking

**Fig. 4:**
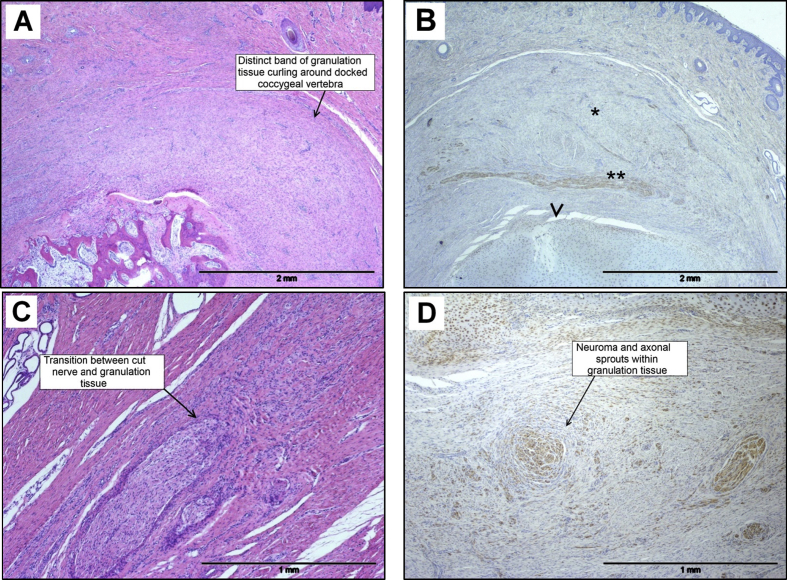
Photomicrographs of histopathological features in sections of pig tail stump 8 weeks after docking. (A) Fully healed superficially with no external signs of tissue trauma associated with amputation. A granulation tissue ‘cap’ is evident in the dermis. HE. (B) Granulation tissue cap (*) covering docked end of coccygeal bone (arrowhead), with peripheral nerve (**) curving around the docked end (S100 expression). IHC. (C) Transition between transected nerve and granulation tissue. HE. (D) Neuroma and multiple axonal sprouts within the granulation tissue (S100 expression). IHC.

On gross examination at 8 weeks of age, the docked tails were fully healed with no obvious external signs of tissue trauma associated with amputation. Many histopathological features were similar to those seen 4 weeks after tail docking. All tails were fully re-epithelialized, with some signs of mild epidermal hyperplasia, orthokeratotic hyperkeratosis and parakeratosis. Mild intra-epidermal and subcorneal pustules were observed in one of the tails. Mild, superficial and deep, perivascular dermatitis was present in some tails (mainly consisting of lymphocytes and plasma cells). In the dermis a well-defined granulation tissue cap was still evident, comprising dermal fibroplasia and angiogenesis, with capping of the cut vertebral end (Fig. 4A, B). Mild, dermal, neutrophilic inflammation was evident in two of four tails, but dermal oedema was not present. Some evidence of bone remodelling was present in one tail, but coccygeal myofibre atrophy or regeneration was not observed. Neuromas/neuromatous tissues were observed in all tails by S100 immunolabelling at this stage post injury (in both dorsal and ventral nerves), with less apparent axonal and nerve sheath proliferation (Fig. 4C, D).

### Histopathology 16 Weeks after Tail Docking

**Fig. 5:**
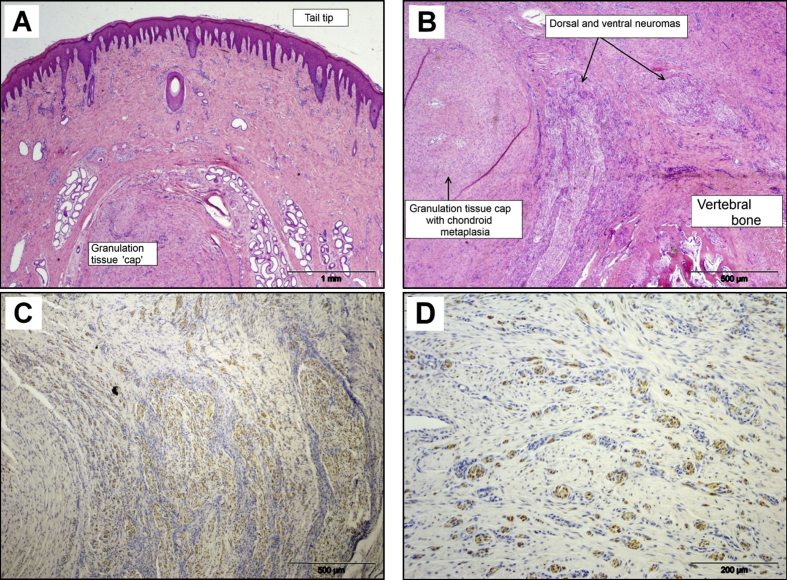
Histopathological features in sections of pig tail stump 16 weeks after docking. (A) Fully healed tail tip with granulation tissue cap over the cut end of the coccygeal bone. HE. (B) Mature granulation tissue ‘cap’ regression with reduced dermal fibroplasia and angiogenesis. HE. (C) Dorsal and ventral neuromas with axonal sprouts in granulation tissue (S100 expression). IHC. (D) Neuroma axonal sprouts dispersed in mature granulation tissue (S100 expression). IHC.

All tails appeared fully healed and were free from minor cuts or abrasions on gross examination. Microscopically, all tails were fully re-epithelialized, with some signs of mild epidermal hyperplasia, orthokeratotic hyperkeratosis and parakeratosis (Fig. 5A). Intra-epidermal or subcorneal pustules were not observed. Mild, superficial, perivascular lymphoplasmacytic inflammation and spongiosis were present in two of four tails. Mild dermal neutrophilic inflammation was evident in two of four tails, but dermal oedema was not present. In the dermis, a granulation tissue cap was still evident in all the tails (although more pronounced in some tails than in others). This was characterized by dermal fibroplasia and angiogenesis that was a little less pronounced compared with earlier time points (Fig. 5B). Similarly, myofibre atrophy was absent, with only limited evidence of myofibre regeneration. S100 immunolabelling revealed mild nerve sheath thickening and moderate axonal proliferation and sprouting in all tails, with widespread axonal infiltration of the superficial tail tip dermis in three of four tails (Fig. 5C). Neuromas of varying sizes and forms (i.e. diffuse and circumscribed) were present in the deep dermis and dispersed in the proximal part of the granulation tissue (Fig. 5D).

### Statistical Analyses

The Fisher Exact test revealed a significantly lower (*P* <0.05) neuroma formation 1 week after tail docking compared with the later time points.

## Discussion

This is the first study to characterize the histological and immunohistochemical features of tail docking injury and repair in neural and non-neural tissue in pigs at several time points during their life. This will help determine the presence and severity of such pathological features and their possible influence on the experience of tail stump pain. This study is the first to report on the time course of traumatic neuroma development in pig tails using S100 neurofilament immunohistochemistry (IHC) of caudal peripheral nerves and subsequent neuromata formation and confirms that traumatic neuroma development and active tail stump re-innervation is still ongoing 16 weeks after tail docking injury. In addition, using S100 IHC, we were also able to describe, for the first time, the different stages of tail stump re-innervation and traumatic neuroma remodelling after tail docking.

One week after tail docking injury the healing of the superficial integumentary layers was almost complete and followed a common pattern of wound healing in man and other mammalian species (Guo and DiPietro, 2010). Beyond this time the injured tail tissues underwent classical proliferative changes accompanied by angiogenesis and transition into a remodelling phase by week 8. In man, this tissue remodelling phase after limb amputation can last up to 2 years (Galiano and Mustoe, 2010). In the present study the pigs were investigated up to slaughter age (i.e. approximately 16 weeks) and it is clear that at 4 months after tail injury, underlying tissue remodelling, specifically in relation to peripheral nerve axonal sprouting, is still ongoing (Fig. 5C). It is not yet fully understood if there is altered tissue sensitivity to external stimuli during the remodelling phase, but it has been reported following the transition from granulation tissue to scar formation, that associated wound contraction can cause abnormal or painful sensations in the affected tissues (Singer and Clark, 1999).

A key concern relating to tail docking injury is the risk of bacteria gaining entry to the wound and progressing deep into the tail tissues, leading to systemic infection. Evaluation of docking methods (e.g. surgical cutters versus hot iron cautery) has been carried out in previous studies (Done et al., 2003, Sutherland et al., 2008, Marchant-Forde et al., 2009), although little difference was found in the relative patterns of healing and secondary infection between methods. In the present study, the presence of surface bacteria was evident in all tails (although identification of the strains was not undertaken). Despite this, there was little or no indication of superficial or deep tissue bacterial infection (Table 1). This may be attributable to the combined effects of tissue sterilization and coagulation produced by thermal cautery.

In human studies of traumatic neuromas and post-amputation pain, patients who had histological signs of chronic inflammation in their biopsy sample (typically a mononuclear cell inflammatory infiltrate), frequently reported symptoms of tingling or pain (Vora *et al.*, 2007), although a causal relationship between histological signs of inflammation and pain or abnormal sensory experience requires further investigation since, in some patients, such symptoms can occur without inflammation. In the present study, little or no superficial or deep tissue inflammation (acute or chronic) was observed after tail injury by thermal cautery, although some evidence of minor ulceration and abscess formation was observed in one tail, 1 week after docking. This suggests that the process of repair, proliferation and remodelling of the tail tissues in this instance progresses without the development of any associated chronic inflammation; therefore, the likelihood of proximal stump pain attributable to inflammation seems low. These findings are consistent with those observed in previous studies on pig tail histopathology (Simonsen et al., 1991, Done et al., 2003).

It would appear that infectious and inflammatory factors may not play a major role in the experience of pain or abnormal sensation in the tail stump after tail docking in pigs 1 week and beyond, although acute short-term inflammatory tail stump pain immediately after docking is likely (Sutherland et al., 2008, Sutherland et al., 2011), even though it was not assessed in this study. It has long been recognized that the regenerating and proliferating nerves in traumatic neuromas can produce non-evoked pain (Wall and Gutnick, 1974). Axons within neuromas can develop abnormal electrical excitability, which is the likely cause of neuroma-associated pain (Devor, 1983, England et al., 1996, England et al., 1998). As the generation of axonal action potentials depends on voltage-gated ion channels, the abnormal electrical activity of neuromas may be the consequence of altered ion channel distribution or properties. Traumatic neuromas in people typically develop approximately 6–10 weeks after surgical nerve injury, gradually enlarging over 2–3 years (Foltán *et al.*, 2008). In man, the majority of patients present with symptoms typically associated with traumatic neuroma development (e.g. paraesthesia, dysaesthesia, neuralgic pain), generally 1–12 months after injury or surgery (Rajput *et al.*, 2012), and these symptoms appear in <10% of patients with post-injuries or surgical traumatic neuromas (Van der Avoort *et al.*, 2013). In the majority of patients with amputation injuries, traumatic neuromas are asymptomatic.

In the present study, neuromatous tissue formation was observed as early as 1 month after tail docking (Table 1) and neuroma development typically progressed consistent with previous reports in pigs (Done *et al.*, 2003) and people (Foltán *et al.*, 2008). Widespread axonal proliferation and infiltration of the superficial dermis was also observed at each time point, suggesting that the process of neuroma formation was still ongoing up to 4 months after docking. During this proliferation phase, peri- and epineurial tissues typically attenuate around proliferating axons as a defence mechanism to protect neural fibres from wound contraction injury, a major factor in post-amputation neuroma-related pain (Foltán *et al.*, 2008). Wound healing in the present study typically progressed by primary intention without signs of infection (except in one tail, 1 week after docking) or abnormal tissue healing. In man, it has been reported that proximal stump pain is more likely to occur under conditions where complicating factors such as infection, haematoma, the presence of foreign bodies, wound irritation and delayed healing are present (Argenyi *et al.*, 1992).

It is currently not known if the stage of maturation of the developing neuroma impacts on possible tail stump sensitivity. It is not possible to confirm, solely on the basis of histopathological assessment, if this adversely affects pig tail stump sensitivity. Further studies, including gene/protein expression analysis of neuropeptide mediators of nociception/pain in neural tissues (e.g. the caudal dorsal root ganglia and spinal cord), currently in progress, are required to attempt to address this question.

In summary, tail docking produces a significant tail injury. The observed histopathological lesions that occur shortly after tail docking (1 week post docking and beyond) are not likely to induce or maintain pain. It is clear that tail docking injury by the hot iron cautery method appears to heal normally without overt signs of secondary intention. The development of traumatic neuromata months after tail docking is a consistent pathological feature with this type of injury, and it would appear that traumatic neuroma development (axonal proliferation and dispersion) is still ongoing up to 4 months after tail docking.

## Figures and Tables

**Table 1:** Histopathological features found in the tail tips of tail docked piglets

Pathological feature	Time after tail docking				Significance^1^	Significance^2^
1 Week	4 Weeks	8 Weeks	16 Weeks	*P*-value	*P*-value
Surface crust/debris	4/4+++	3/4+	2/4+	3/4+		
Surface bacteria	4/4+++	3/4+	3/4+	4/4+		
Hyperkeratosis (orthokeratotic)	1/4+	4/4+++	4/4+	4/4+++		
Parakeratosis	2/4+	3/4+	4/4+	2/4+		
Epidermal hyperplasia	4/4+++	4/4++	4/4++	4/4++		
Spongiosis	3/4++	0	0	2/4+		
Anastomosing rete pegs	3/4+++	4/4++	4/4++	4/4++		
Subcorneal pustules∗	1/4+++	0	1/4+	0		
Intra-epidermal pustules∗	1/4+	1/4+	1/4+	0		
Full re-epithelialization	1/4	4/4	4/4	4/4		
Epidermal erosion∗	2/4+	0	0	0		
Ulceration∗	2/4+++^a^	0^b^	0^b^	0^b^	0.046	<0.05
Superficial perivascular inflammation	2/4++	1/4+	2/4+	2/4+		
Deep perivascular inflammation	1/4++	0	1/4+	0		
Dermal oedema∗	4/4+^a^	0^b^	0^b^	0^b^	0.013	<0.05
Fibroplasia	3/4+++	4/4+++	4/4+++	4/4+++		
Dermal angiogenesis	4/4+++	4/4++	4/4++	2/4++		
Granulation tissue	4/4+++	4/4+++	4/4+++	4/4+++		
Thrombosis	1/4+	0	0	0		
Dermal neutrophilic inflammation∗	4/4+++	2/4++	2/4+	2/4+		
Cellulitis∗	1/4+	0	0	0		
Osteomyelitis∗ (where bone was present)	0	0	0	0		
Bone remodelling (where bone was present)	2/4+	0	1/4+	0		
Myofibre atrophy	4/4++	1/4+	0	0		
Myofibre regeneration	3/4+	1/4+	0	1/4+		
Nerve/axonal proliferation†	3/4++	4/4++	4/4++	4/4++		
Neuroma/neuromatous tissue†	0^a^	2/4++^a^	4/4++^b^	4/4++^b^	0.011	<0.05
Axonal infiltration of superficial dermis†	4/4+	4/4++	4/4++	3/4+		

Data values represent the number of pigs at each time point after tail docking exhibiting that feature. + symbols represent the maximum observed abundance score (+, low; ++, medium; +++, high) of four pigs at that time point after docking. Significance^1^ shows comparison of prevalence of that feature for tail docking (TD) + 1 week against all later time points (Fisher's Exact test: same superscripts do not differ significantly). Significance^2^ shows comparison of the maximum abundance scores for TD + 1 week against all later time points (Mann–Whitney *U* test).

∗ Features deemed likely to induce or maintain pain.

† Identified by S100 IHC.
