# Corrigendum to “Histopathological Characterization of Tail Injury and Traumatic Neuroma Development after Tail Docking in Piglets” [J Comp Pathol 155 (1) (2016) 40–49]

**DOI:** 10.1016/j.jcpa.2016.08.002

**Published:** 2016

**Authors:** D.A. Sandercock, S.H. Smith, P. Di Giminiani, S.A. Edwards

**Affiliations:** ∗ Animal and Veterinary Science Research Group, Scotland's Rural College (SRUC), West Mains Road, Edinburgh, UK; † Royal (Dick) School of Veterinary Studies and the Roslin Institute, Easter Bush Campus, Roslin, Midlothian, UK; ‡ School of Agriculture, Food and Rural Development, Newcastle University, Newcastle upon Tyne, UK

The authors wish to clarify terminology used in their paper entitled ‘Histopathological characterization of tail injury and traumatic neuroma development after tail docking in piglets’ and thank the Editor for the opportunity to do so. In the absence of a specific immunohistochemical label for detection of axons, the words ‘axon/axonal’ were inaccurately used and should be replaced by ‘Schwann cell’. Without more specific proof, it certainly does not confirm, or necessarily infer, conduction. Secondly, ‘S100 neurofilament’ was inadvertently used instead of simply ‘S100’. The authors apologise for this error, which was wholly editorial on their part. Finally, in our opinion, the literature definitions of traumatic neuromas are such that there is likely to be some disagreement as to their required component features, particularly at different stages of lesion development, in different species and in different age groups of animals. In our paper, descriptions of traumatic neuroma presence and development were also based on haematoxylin and eosin staining and not solely confined to S100 immunolabelling. To this end, features such as variably-sized microfascicles, disorderly (often circumferential) neural proliferation and nerve fibres turning back on themselves are consistent with previous reports on traumatic neuromas in a number of species, including pigs.

While the aforementioned errors are regretted, this work was intended as a descriptive morphological characterization of a wide range of histopathological changes over known time points post docking. We have had some criticism that, due to our use of the word axonal, we have implied or claimed innervation, and thus pain sensation, during the weeks after docking. This was not our intention – rather, our opinion is neutral in terms of whether or not traumatic neuromas are painful. The last sentence of the paper acknowledged that this work could not determine that. This study was considered descriptive and foundational, to serve as a platform for further investigation. Its take-home message, irrespective of the error in terminology, is that neural proliferation consistent with traumatic neuroma development appears to be still ongoing at 16 weeks after tail docking.

