# Supplementary material for: Histopathological Characterization of Tail Injury and Traumatic Neuroma Development after Tail Docking in Piglets
Source: J Comp Pathol. 2016 Jul;155(1):40–9. doi: 10.1016/j.jcpa.2016.05.003 (PMC4940206; doi:10.1016/j.jcpa.2016.05.003)
Supplement: Supplementary file 1 [file mmc1.docx]

| **Pathological feature** | **Relative abundance (score)** | | |
| --- | --- | --- | --- |
|  | **Low (+)** | **Medium (++)** | **High (+++)** |
| Surface crust/debris | Intermittent crust < epidermal thickness | Tail tip covered in thin crust (< epidermal thickness) | Tail tip covered with thick crust (> epidermal thickness) |
| Surface bacteria | Light colonisation of crust | Moderate colonisation of crust (~5-10 colonies per tail) | Heavy colonisation of crust (>10 colonies per tail) |
| Hyperkeratosis (orthokeratotic) | < Half epidermal thickness | > half but < full epidermal thickness | Thicker than the epidermis, even if focal |
| Parakeratosis | Small localised focus/foci | Diffuse but < epidermal thickness | Thicker than epidermis even if focal |
| Epidermal hyperplasia | Up to 1.5 times normal epidermal thickness | > 1.5 but < twice normal epidermal thickness | > twice normal epidermal thickness |
| Spongiosis | Subtle expansion of intercellular space even if focally | Clearly visible intercellular spaces but not diffuse | Diffuse clearly visible intercellular space |
| Anastomosing rete pegs | Mild lengthening beyond normal | Extension to mid dermis | Extension beyond superficial dermis with anastomoses |
| Subcorneal pustules | Single pustule spanning no more than one follicle length | Single pustule spanning > two follicles | Single pustule spanning > three follicles or multiple pustules |
| Intra-epidermal pustules | Single pustule spanning no more than one follicle length | Single pustule spanning > two follicles | Single pustule spanning > three follicles or multiple pustules |
| Full re-epithelialisation | Yes/no | | |
| Epidermal erosion | Loss of most superficial keratinocytes | Half thickness epidermal loss | Almost full thickness epidermal loss but no breaching of the basement membrane |
| Ulceration | Focal punctate full thickness epidermal loss | Full thickness epidermal loss affecting up to half the tail tip | Full thickness epidermal loss affecting > half the tail tip |
| Superficial perivascular inflammation | Rare lymphocytes, plasma cells, neutrophils or eosinophils | At least one perivascular focus of inflammatory cells (>10 cells) | Multiple vessels each surrounded by >10 inflammatory cells |
| Deep perivascular inflammation | Rare lymphocytes, plasma cells, neutrophils or eosinophils | At least one perivascular focus of inflammatory cells (>10 cells) | Multiple vessels each surrounded by >10 inflammatory cells |
| Dermal oedema | Increased pallor in superficial dermis | Increased pallor from superficial to mid dermis | Diffuse increased dermal pallor |
| Fibroplasia | Mild increase in loosely spaced fibroblasts in dermis and/or subcutis | Moderate increase fibroblasts in dermis and/or subcutis | Marked increase in fibroblasts in dermis and/or subcutis |
| Dermal angiogenesis | Slight increase in dermal capillaries above normal level | Moderate increase in density of dermal capillaries | Marked increase in dermal capillaries |
| Granulation tissue | Focal granulation tissue anywhere in the dermis or subcutis | Granulation tissue effacing tail tip dermis/subcutis | Granulation tissue expanding tail tip and/or lateral dermis/subcutis |
| Thrombosis | Solitary thrombus anywhere in section | <5 thrombi in section | >5 thrombi in section |
| Dermal neutrophilic inflammation | Rare neutrophils | At least one focus of neutrophils (>10 cells) | Multiple foci of neutrophils (>10 cells) |
| Cellulitis | Single focus of inflammatory cells in subcutis only | Multiple foci of inflammatory cells in subcutis | Regionally extensive or multifocal to coalescing foci of inflammatory cells in subcutis |
| Osteomyelitis | Inflammatory cells in vertebral marrow space affecting <10% of marrow | Inflammatory cells in vertebral marrow space affecting 10-50% of marrow | Inflammatory cells in vertebral marrow space affecting >50% of marrow |
| Bone remodelling | Single focus of osteoclasts (<5 osteoclasts) | <3 clusters of osteoclasts (<5 osteoclasts per cluster) | >3 clusters of osteoclasts or <3 clusters but each with >5 osteoclasts |
| Myofibre atrophy | Rare myofibres reduced in diameter | Multifocal small clusters of myofibres reduced in diameter | Regionally extensive or diffuse reduction in myofibre diameter |
| Myofibre regeneration | <5 regenerating myofibres per section | 5-10 regenerating myofibres per section | >10 regenerating myofibres per section |
| Nerve/axonal proliferation | Mild or localised increase in axonal sprouts within dermis/subcutis | Moderate or more widespread increase in axonal sprouts within dermis/subcutis | Marked increase in axonal sprouts within dermis/subcutis |
| Neuroma | Mild or localised increase in disorganised axonal sprouts within granulation tissue | Moderate or more widespread increase in disorganised axonal sprouts within granulation tissue | Marked increase in disorganised axonal sprouts within granulation tissue |
